# Mechanisms of ozone-induced neurotoxicity in the development and progression of dementia: a brief review

**DOI:** 10.3389/fnagi.2024.1494356

**Published:** 2024-10-28

**Authors:** Luis A. Marin-Castañeda, Guillermo Gonzalez-Garibay, Isabella Garcia-Quintana, Gerónimo Pacheco-Aispuro, Carmen Rubio

**Affiliations:** ^1^Department of Neurophysiology, Instituto Nacional de Neurología y Neurocirugía “MVS”, Mexico City, Mexico; ^2^Facultad de Medicina, Universidad Nacional Autónoma de México, Mexico City, Mexico; ^3^Hospital Ángeles del Pedregal, Mexico City, Mexico

**Keywords:** ozone, neurodegeneration, neuroinflammation, dementia, Alzheimer’s disease

## Abstract

Dementia encompasses a spectrum of neurodegenerative disorders significantly impacting global health, with environmental factors increasingly recognized as crucial in their etiology. Among these, ozone, has been identified as a potential exacerbator of neurodegenerative processes, particularly in Alzheimer’s disease (AD). Ozone exposure induces the production of reactive oxygen species (ROS), which penetrate the BBB, leading to oxidative damage in neuronal cells. This oxidative stress is closely linked with mitochondrial dysfunction and lipid peroxidation, processes that are foundational to the pathology observed in dementia, such as neuronal death and protein aggregation. Furthermore, ozone triggers chronic neuroinflammation, exacerbating these neurodegenerative processes and perpetuating a cycle of CNS damage. Recent studies highlight the role of peripheral biomarkers like High Mobility Group Box 1 (HMGB1) and Triggering Receptor Expressed on Myeloid cells 2 (TREM2) in mediating ozone’s effects. Disruption of these and other identified proteins by ozone exposure impairs microglial function and response to amyloid plaques, suggesting a novel pathway through which ozone may influence AD pathology via immune dysregulation. This review discusses the concept of a bidirectional lung-brain axis, illustrating that systemic responses to air pollutants like ozone may reflect and contribute to neurodegenerative processes in the CNS. By delineating these mechanisms, we emphasize the critical need for integrating environmental health management into strategies for the prevention and treatment of dementia.

## 1 Introduction

Dementia encompasses a broad range of neurodegenerative disorders that represent a growing global health crisis. Currently, fifty million people worldwide live with a form of dementia, a number that is expected to rise sharply as populations age (Gale et al., 2018; Chandra et al., 1994). The most common types of dementia, such as Alzheimer’s disease (AD), vascular dementia (VaD), Lewy body dementia, and frontotemporal dementia, cause severe cognitive impairment and memory loss, significantly impacting patients’ quality of life (Raz et al., 2016). These disorders result from a complex interaction of genetic, environmental, and lifestyle factors (Carey et al., 2018; Fiala et al., 2007; Kunkle et al., 2019).

Significant progress has been made in understanding the genetic and biological underpinnings of dementia, increasing attention is being directed toward the role of environmental factors, particularly air pollution. Among the various pollutants, ground-level ozone (O3) has emerged as a critical concern due to its pervasive presence and potential to contribute to neurodegenerative processes (Singh et al., 2022). O3 is formed in the troposphere through multiple photochemical reactions involving nitrogen oxides and volatile organic compounds, both of which are primarily emitted through human activities such as industrial processes and vehicular emissions (Martínez-Lazcano et al., 2013).

The widespread exposure to ozone and other air pollutants is alarming. According to the World Health Organization, approximately 99% of the global population breathes air that exceeds recommended quality standards, with urban populations being particularly at risk (World Health Organization, 2024; American Lung Association, 2024; World Health Organization, 2021). In the United States, 39% of the population lives in areas with air pollution levels high enough to pose significant health risks, according to the American Lung Association’s (2024) “State of the Air” report. These statistics underscore the urgent need to recognize the health impacts of air pollutants, particularly their potential role in initiating and exacerbating neurodegeneration (American Lung Association, 2024).

The central nervous system (CNS) is particularly vulnerable to oxidative stress, a condition where the production of reactive oxygen species (ROS) overwhelms the antioxidant defenses, leading to cellular damage. Ozone exposure is a powerful inducer of oxidative stress, with the ability to generate ROS that penetrate the blood-brain barrier (BBB). This penetration can lead to lipid peroxidation, protein oxidation, and mitochondrial dysfunction, processes that are intimately linked with the pathological events observed in dementia, such as neuronal death and protein aggregation (Singh et al., 2022; Kunkle et al., 2019). Moreover, ozone-induced oxidative stress can trigger chronic neuroinflammation and further compromise the BBB, creating a vicious cycle that exacerbates neurodegenerative processes.

Increasing evidence suggests an environmental influence in dementia etiology, with various components of air pollution now linked to an elevated risk of neurodegeneration (Croze and Zimmer, 2018; Greve et al., 2023; Jayaraj et al., 2017; Kilian and Kitazawa, 2018). The aim of this paper is to examine the potential role of ground-level ozone as a contributing factor to neurodegenerative processes, particularly in relation to dementia. By reviewing the latest evidence on O_3_ exposure, oxidative stress, and the central nervous system, thus contributing to the broader understanding of environmental factors in dementia etiology.

## 2 Oxidative stress and neuronal damage

Reactive oxygen species refers to a diverse group of oxygen-containing molecules that are highly reactive. These include free radicals and their derivatives, which are known to initiate oxidative transformations within cells. In healthy tissues, ROS are naturally produced across various cellular locations as part of standard metabolic processes (Migliore and Coppedè, 2009; Zhang et al., 2008). The primary sites where ROS are formed within the cell is in the mitochondrial electron transport chain, specifically at complex I (NADH dehydrogenase) and complex III (ubiquinone-cytochrome c reductase). Other major sources of ROS production within the body include: peroxisomal fatty acid metabolism, reactions involving cytochrome P-450, oxidative bursts from phagocytic cells, and several complex enzymatic pathways (Harman, 1992; Huang and Manton, 2004; Lima et al., 2022; Sies et al., 2024). While ROS are essential for maintaining oxygen equilibrium in tissues and combating pathogens, they are also capable of inducing oxidative modifications to cellular constituents, affecting proteins, lipids, and nucleic acids structures. These changes are linked with the development and progression of age-related conditions (Chakravarti and Chakravarti, 2007; Sakai et al., 2014; Schieber and Chandel, 2014).

To counteract ROS-induced damage, cells deploy a variety of antioxidant mechanisms, including enzymes, vitamins, and other metabolites. These protect the cell through three main strategies: neutralizing ROS and their precursors, binding catalytic metals that facilitate ROS production, and bolstering the cell’s inherent defense systems. The balance between ROS production and the effectiveness of the antioxidant defense determines the extent of oxidative stress. If ROS production overwhelms the antioxidant defense capabilities, oxidative stress ensues, leading to significant damage to vital macromolecules along with mitochondrial and other cellular structures (Joseph et al., 2005; Patel, 2016; Salim, 2017). It has been demonstrated that repeated exposure to low-ozone doses leads to a deterioration in the response of antioxidant mechanisms, therefore causing a chronic oxidative stress state (Velázquez-Pérez et al., 2021).

Moreover, aging can be understood as a gradual accumulation of damage to macromolecules, primarily from free radicals originating in mitochondria. The vulnerability of mitochondrial DNA is due to several factors: its proximity to the sites of ROS generation, the absence of a complete histone protective coat around this organelle, and inadequate repair mechanisms (Huang and Manton, 2004; Migliore and Coppedè, 2009). Over time, as individuals age, the damage inflicted by free radicals on mitochondria increases, ROS production rises, and the cell’s antioxidant defenses gradually weaken. This growing susceptibility to oxidative stress contributes to the aging process and related degenerative conditions (Frijhoff et al., 2015; Genestra, 2007; Phaniendra et al., 2015).

While cells that replicate quickly and have low oxygen requirements are less prone to such damage, highly differentiated cells like neurons, with high oxygen demands, are significantly at risk (Zhang et al., 2011; Zhang et al., 2008). This vulnerability of neurons to oxidative stress is particularly notable because damaged cells cannot be easily replaced, thus amplifying the effects of aging. The role of ozone is crucial in this context, as it directly contributes to neuronal oxidative stress through molecular pathways that increase ROS production (Pereyra-Muñoz et al., 2006). When ozone enters the respiratory system, it undergoes enzymatic reactions in the pulmonary lining fluids and generates a series of reactive oxygen species, including hydrogen peroxide, hydroxyl radicals, and superoxide anions (Kilburg-Basnyat et al., 2018). These can diffuse into the bloodstream and reach the central nervous system, where they further interact with cellular components, disrupting mitochondrial electron transport chains. This disruption leads to an inefficient electron flow in mitochondria, causing electron leakage and further ROS production at complexes I and III. Moreover, ozone exposure can activate NADPH oxidases, which are enzymes directly responsible for producing superoxide radicals as part of the cellular response to environmental stressors. Ozone can cause the depletion of NAD+ cellular reserves, leaving the neuron susceptible to molecular insults (Ma et al., 2020).

Enhanced ROS levels within neurons lead to oxidative modifications of lipids, proteins, and DNA, triggering cellular repair mechanisms which can be overwhelmed if oxidative stress is persistent. Experimental studies, such as those by Kodavanti et al. (2021), demonstrate that sub-chronic ozone exposure results in increased cellular damage, evidenced by heightened protein carbonyl content and reduced aconitase activity, both indicative of oxidative stress and mitochondrial DNA destabilization. This condition further triggers an enhanced influx of calcium into cells, exacerbating neuronal damage, and facilitating the release of ATP through exocytosis, which impacts purinergic receptors, a process also detailed by Velázquez-Pérez et al. (2021). Such biochemical changes are accompanied by the activation of enzymes like NADPH quinone oxidoreductase 1 (NQO1) and NADH ubiquinone reductase (UBIQ-RD), catalyzing redox reactions that further contribute to the neurodegenerative process. Additionally, increased calcium influx and a decrease in succinate dehydrogenase activity significantly contribute to cell death, as noted by Rodríguez-Martínez et al. (2016).

It has been suggested that hippocampal cells in the brain are among the most affected by oxidative stress. The significant calcium influx, resulting from various biochemical pathway alterations, leads to endoplasmic reticulum damage and subsequent protein misfolding (Nishida et al., 2012; Rodríguez-Martínez et al., 2013, 2016). Additionally, the damage to the mitochondria promotes a decrease in the levels of available ATP in the cell, shifting the cellular metabolism and modifying the internal environment due to failure of ATP-dependent pumps. This leads to increased extracellular concentrations of ATP which acts in purinergic receptors and changing the dynamics of intracellular signaling pathways related to cell survival.

Previous research has underscored the significant role of P2X7 receptors in neuronal dynamics. Specifically, stimulation of these receptors results in the phosphorylation of GSK3β at serine 9, which influences neuronal glycogen levels by altering glutamyl synthetase enzyme activity (Brookes et al., 2004; Martin et al., 2018). Dysfunction of P2X7 receptors has been observed during chronic oxidative states, leading to significant biochemical disruptions (Volonté et al., 2003). This dysfunction inhibits GSK3β, resulting in decreased disposal of glucose 6 phosphate and causing allosteric dysregulation of glucose and glycogen metabolism, ultimately increasing neuronal death (Ferrari et al., 1997; Pelegrin, 2021; Mergenthaler et al., 2013; Velázquez-Pérez et al., 2021).

The progressive impairment of neurons through oxidative damage is closely linked to the development of cognitive impairments and dementias. As the brain ages, the cumulative effect of this damage can lead to significant functional decline, manifesting as various forms of dementia (Ahmad et al., 2022; Tönnies and Trushina, 2017).

## 3 Neuroinflammatory cascade

Neuroinflammation is a recognized consequence of neurodegeneration in numerous chronic neurological disorders, such as major depressive disorder (Frank et al., 2021), multiple sclerosis (Zelic et al., 2021), amyotrophic lateral sclerosis (Beers and Appel, 2019), Parkinson’s disease (Sampson et al., 2016), and Huntington’s disease (Quinn et al., 2020). Furthermore, it has recently been implicated in the progression of Alzheimer’s disease and other neurodegenerative disorders (Mayne et al., 2020). Emerging evidence supports the neuroinflammation hypothesis, which suggests that proinflammatory markers in the CNS, driven by the activation of resident immune cells like microglia, play a crucial role in the development of dementia.

**FIGURE 1:**
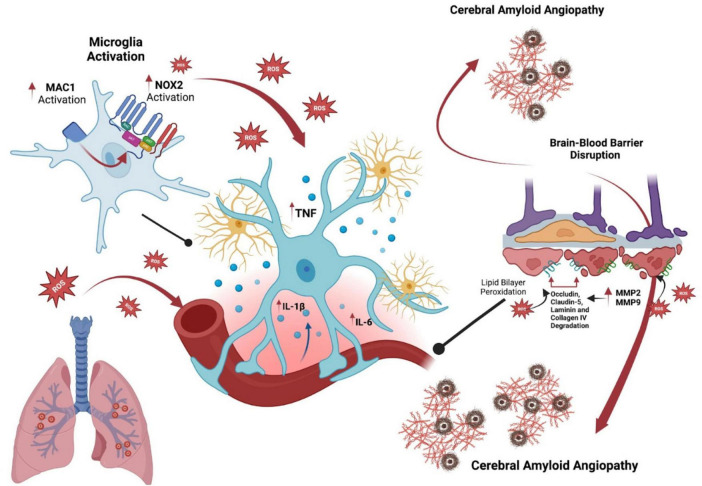
Influence of ozone on microglial activation and its role in cerebral amyloid angiopathy and Alzheimer’s disease. This diagram illustrates the role of ozone exposure in microglial activation and its implications for Alzheimer’s disease via cerebral amyloid angiopathy. Initially, ozone induces microglial cells to activate, increasing NOX2 activity and reactive oxygen species (ROS) production. These oxidative agents prompt glia to release pro-inflammatory cytokines such as TNF, IL-1β, and IL-6, contributing to neuroinflammation and neuronal distress. The right part of the image highlights the chronic activation of microglia, accelerating amyloid plaque deposition in cerebral vessels, which is a hallmark of cerebral amyloid angiopathy in Alzheimer’s disease. This progression disrupts the blood-brain barrier through the activation of MMP2 and MMP9, leading to the degradation of structural proteins like occludin, claudin-5, laminin, and collagen IV. This sequence emphasizes the pathways through which environmental factors such as ozone may exacerbate Alzheimer’s disease through neurovascular and inflammatory mechanisms.

It is well established that the neuroinflammatory process is characterized by the release of cytokines, including IL-1β, IL-6, IL-18, and tumor necrosis factor (TNF), as well as chemokines such as C-C motif chemokine ligand 1 (CCL1), CCL5, and C-X-C motif chemokine ligand 1 (CXCL1). Additionally, small-molecule messengers like prostaglandins and nitric oxide (NO), along with ROS, are produced by innate immune cells within the CNS. These factors are believed to play a causal role in progressive neuronal and myelin damage (Block et al., 2007; Jayaraj et al., 2017; Figure 1).

Microglia are the brain’s resident innate immune cells, essential for maintaining CNS health (Hong et al., 2016; Reemst et al., 2016; Solé-Domènech et al., 2016). However, when their response becomes dysregulated, they can be reprogrammed to produce elevated levels of neurotoxic cytokines, leading to toxicity in surrounding cells, such as neurons and oligodendrocytes (Block et al., 2007; Brown and Vilalta, 2015).

There is ongoing debate about how ozone and other air pollution particles impact the brain, leading to microglia activation and neuroinflammation. The proposed pathways for this activation often involve peripheral effects that influence the brain. Microglia are well known for their ability to detect and respond to these peripheral disturbances, with the production of circulating cytokines being a key mechanism hypothesized to induce neuroinflammation (Figure 1).

However, studies in animal models have shown that microglial activation and CNS effects in response to urban air pollution and ozone can occur both with (Levesque et al., 2011; Rodríguez-Quintero et al., 2024) and without (Mumaw et al., 2016) the involvement of traditional circulating cytokines. This suggests that the neuroinflammatory response may be driven by multiple mechanisms rather than a single pathway. One such mechanism could be neurogenic inflammation, where the activation of peripheral neurons leads to the release of neuropeptides and other inflammatory mediators that contribute to inflammation. Additionally, this process might involve a neurohormonal stress response, further linking peripheral and central mechanisms.

In this context, a lung-brain axis has been proposed, suggesting that microglia can detect and respond to circulating signals, independent of cytokines, that arise from pulmonary injury due to inhaled ozone (Jayaraj et al., 2017; Mumaw et al., 2016). Preclinical studies indicate that ozone exposure activates microglia through unidentified circulating signals related to pulmonary injury, mediated by the microglial antigen complex (MAC). This persistent microglial activation, observed *in vivo*, is accompanied by circulating factors in the serum that enhance proinflammatory microglial activation and amyloid-beta (Aβ) neurotoxicity in *ex vivo* cultures (Mumaw et al., 2016).

Toll-like receptor (TLR) signaling pathways play a crucial role in initiating acute neuroinflammation. Notably, the upregulation of TLRs, particularly TLR2 and TLR4, has been observed in various chronic neurodegenerative conditions, including AD (Gambuzza et al., 2014; Landreth and Reed-Geaghan, 2009). Despite this, the mechanisms by which unresolved, low-grade chronic neuroinflammation contributes to progressive neurodegeneration remain poorly understood. Some studies suggest that while TLR4 is essential for initiating acute neuroinflammation, it may not be sufficient to sustain the chronic inflammatory response.

In this context, the MAC1/NOX2 signaling pathway has garnered increasing attention for its role in the maintenance of chronic neuroinflammation. MAC1 (CD11b/CD18), is a receptor complex expressed on microglia and other immune cells in the CNS. Upon activation, MAC1 interacts with various ligands, leading to the activation of NADPH oxidase 2 (NOX2), an enzyme complex responsible for producing ROS which not only cause direct neuronal damage but also perpetuate microglial activation (Chen et al., 2016; Gao et al., 2012). A critical aspect of this pathway is the persistent activation of extracellular signal-regulated kinases 1 and 2 (ERK1/2).

The sustained activation of ERK1/2 is closely associated with MAC1-linked NOX2 activity and is a key factor in maintaining the reactive state of microglia. ERK1/2 activation leads to the phosphorylation of p47phox, a subunit of NOX2, initiating the translocation of NOX2’s cytosolic subunits to the membrane, which amplifies ROS production. This persistent ERK1/2 activation, observed in wild-type but not in MAC1 knockout microglia, contributes to a self-propelling cycle of ROS production and microglial activation, driving chronic neuroinflammation. Inhibition of ERK1/2 has been shown to disrupt this cycle, reducing microgliosis and protecting neurons from inflammation-induced damage.

In recent investigations, the role of peripheral High Mobility Group Box 1 (HMGB1) has been examined as one of the multiple pathways through which ozone impacts CNS (Greve et al., 2023). Ozone exposure has been demonstrated to elevate levels of this mediator, which is significantly linked with the dysregulation of Triggering Receptor Expressed on Myeloid cells 2 (TREM2) (Roussos et al., 2015). Such impaired TREM2 expression is critical because it plays a pivotal role in the microglial response to amyloid plaques, a hallmark of AD pathology (Greve et al., 2023). This study shows that ozone exposure exacerbates amyloid plaque accumulation and neuritic dystrophy, operating in a manner that mimics the loss of TREM2 function. This suggests that not only does ozone influence central neurodegenerative processes directly, but peripheral responses to ozone, such as the increase in HMGB1 levels, can also mediate these effects by disrupting TREM2 functionality. This bidirectional communication in the lung-brain axis underscores the complexity of neurodegenerative diseases and the influence of systemic factors, suggesting that the peripheral immune system’s response to air pollutants might reflect and contribute to the central neurodegenerative processes. Such insights call for a more integrative approach to understanding and treating neurodegenerative diseases, considering both environmental exposures and their broader biological impacts.

## 4 Blood-brain barrier disruption

The BBB is an endothelial structure that regulates the selective passage of molecules from the general circulation into the brain’s microenvironment (Pryor, 1992). Ozone contributes to BBB disruption indirectly by promoting inflammatory processes. This occurs because ozone upon disintegration in the airways, leads to the production of phospholipases and hydrogen peroxide, highly cytotoxic substances that trigger the release of proinflammatory cytokines (Cooper et al., 2010; Pryor et al., 1995; Uhlson et al., 2002).

The cellular lipid bilayers of the BBB are particularly susceptible to peroxidation by ROS. The degradation of these lipid membranes leads to the formation of 4-hydroxynonenal and malondialdehyde, both of which are markers of lipid peroxidation (Park et al., 2021). ROS also enhance the NF-κB pathway, promoting the transcription of proinflammatory cytokines such as TNF-α, IL-1β, and IL-6 (Yi et al., 2020). These cytokines can further compromise the endothelial barrier by disrupting the molecular structures of key tight junction proteins, including occludin, claudin-5, and zonula occludens-1 (Yan et al., 2023). Such disruptions to the BBB can facilitate the transmigration of leukocytes, thereby exacerbating inflammation (Muller, 2003; Tietz and Engelhardt, 2015).

Matrix metalloproteinases 2 (MMP-2) and 9 (MMP-9) are upregulated by ROS generated following ozone exposure (Galis et al., 1994; Vecil et al., 2000). These enzymes are closely associated with the degradation of occludin, claudin-5, laminin, and collagen IV, which are essential proteins for maintaining BBB integrity and functionality. The overexpression of MMPs and the consequent disruption of the BBB have been linked to cerebral amyloid angiopathy, which is present in approximately 95% of AD patients, particularly in the parietal and frontal lobes (Jellinger, 2002; Maia et al., 2006). These hemorrhages have been shown to exacerbate cognitive decline (Goos et al., 2009). MMPs are upregulated around hemorrhagic areas in amyloid-affected vessels, suggesting their role in BBB breakdown, inflammation, and tau aggregation (Hernandez-Guillamon et al., 2012), (Yin et al., 2006). MMP-9 may also degrade nerve growth factor, potentially contributing to the vulnerability of cholinergic neurons and cognitive decline in AD (Iulita and Cuello, 2016).

Moreover, elevated levels of serum amyloid A (A-SAA) in response to ozone exposure can cross the compromised BBB and contribute to neuroinflammation (Pryor, 1992). A-SAA has been shown to promote the aggregation of Aβ and tau proteins, key events in the progression of AD. The deposition of A-SAA in the frontal cortex, particularly within myelin sheaths, suggests a direct role in the demyelination and synaptic dysfunction observed in dementia (Erickson et al., 2017).

## 5 Conclusion

The concept of a bidirectional lung-brain axis highlights the complex interaction between peripheral organ systems and the brain in response to environmental insults. This axis highlights that the effects of air pollutants like ozone are not confined to direct exposure pathways but also involve systemic responses that can exacerbate or perhaps initiate processes underlying neurodegeneration.

Understanding the mechanisms by which O_3_ contributes to neurodegeneration offers significant implications for public health and policy. Future research should focus on identifying the molecular mediators at the lung-brain axis that are affected by O_3_ exposure. Additionally exploring the efficacy of existing neuroprotective strategies could lead to the development of targeted interventions to mitigate the impact of air pollutants on neurodegenerative processes.
